# SYSTEMATIC EVALUATION OF ADAPTATIONS AND FIDELITY IN PRAGMATIC NURSING HOME CLINICAL TRIALS

**DOI:** 10.1093/geroni/igae098.1374

**Published:** 2024-12-31

**Authors:** Michelle Hilgeman, Caroline Madrigal, Kimberly Curyto, Corilyn Ott, Sophia Geisser, Christine Hartmann, A Lynn Snow

**Affiliations:** Tuscaloosa VA Medical Center, Tuscaloosa, Alabama, United States; VA Boston Healthcare System, Brockton, Massachusetts, United States; Western New York VA Healthcare System, Batavia, New York, United States; Birmingham VA Medical Center, Birmingham VA Medical Center, Alabama, United States; University of Alabama, Tuscaloosa, Alabama, United States; VA Bedford Healthcare System & University of Massachusetts Lowell, Bedford, Massachusetts, United States; University of Alabama, Tuscaloosa, Alabama, United States

## Abstract

Adaptation of interventions to local contexts is often required for sustainable implementation. Yet maintaining and measuring fidelity to an intervention’s core components is necessary during pragmatic clinical trials. We systematically addressed this inherent tension across two studies of Montessori approaches for person-centered care in Department of Veterans Affairs (VA) nursing homes. We first conducted a 3-phase pre-implementation study (2018-2021) to adapt Montessori for VA with input from operations partners, frontline clinical teams, long-term care residents, implementation scientists, and intervention developers. We used the Framework for Reporting Adaptations and Modifications-Enhanced (FRAME) to track changes in real-time using qualitative and quantitative data at eight VAs. Primary motivators for adaptation were expanding reach of the intervention and engagement with staff training/delivery. Next, the adapted Montessori intervention was tested in a pragmatic, randomized stepped-wedge clinical trial at eight additional VAs (2021-2023). Diverse frontline staff (e.g., nursing assistants, nurses, therapists) delivered the intervention to all residents. Low burden methods were used to gather reach, implementation, and fidelity data over 12-months (i.e., 15-minute weekly neighborhood check-ins, individual staff surveys, organizational-level fidelity tools completed by the clinical team). Adherence to and delivery of the intervention remained high at most sites over time across all measures. Evidence of fidelity and sustained use across sites in the trial may be attributed to the fit of the adapted Montessori intervention with the needs, resources, and clinical priorities of VA long-term care residents and staff.

